# Safety and enhanced immunostimulatory activity of the DRD2 antagonist ONC201 in advanced solid tumor patients with weekly oral administration

**DOI:** 10.1186/s40425-019-0599-8

**Published:** 2019-05-22

**Authors:** Mark N. Stein, Jyoti Malhotra, Rohinton S. Tarapore, Usha Malhotra, Ann W. Silk, Nancy Chan, Lorna Rodriguez, Joseph Aisner, Robert D. Aiken, Tina Mayer, Bruce G. Haffty, Jenna H. Newman, Salvatore M. Aspromonte, Praveen K. Bommareddy, Ricardo Estupinian, Charles B. Chesson, Evita T. Sadimin, Shengguo Li, Daniel J. Medina, Tracie Saunders, Melissa Frankel, Aparna Kareddula, Sherrie Damare, Elayne Wesolowsky, Christian Gabel, Wafik S. El-Deiry, Varun V. Prabhu, Joshua E. Allen, Martin Stogniew, Wolfgang Oster, Joseph R. Bertino, Steven K. Libutti, Janice M. Mehnert, Andrew Zloza

**Affiliations:** 1 Division of Hematology/Oncology, Columbia University Medical Center, New York, NY USA; 2 Rutgers Cancer Institute of New Jersey, Rutgers, The State University of New Jersey, New Brunswick, NJ USA; 3 Oncoceutics, Inc, Philadelphia, PA USA; 4 Department of Dermatology and Department of Medicine, Division of Medical Oncology, Dana-Farber Cancer Institute, Boston, MA USA; 5 Warren Alpert Medical School, Brown University, Providence, Rhode Island USA; 6 Division of Hematology, Oncology, and Cell Therapy, Department of Internal Medicine, Rush University Medical Center, Chicago, IL USA

**Keywords:** ONC201, Cancer, Solid tumors, Immunotherapy, Immuno-oncology, Dopamine

## Abstract

**Background:**

ONC201 is a small molecule antagonist of DRD2, a G protein-coupled receptor overexpressed in several malignancies, that has prolonged antitumor efficacy and immunomodulatory properties in preclinical models. The first-in-human trial of ONC201 previously established a recommended phase II dose (RP2D) of 625 mg once every three weeks. Here, we report the results of a phase I study that evaluated the safety, pharmacokinetics (PK), and pharmacodynamics (PD) of weekly ONC201.

**Methods:**

Patients ≥ 18 years old with an advanced solid tumor refractory to standard treatment were enrolled. Dose escalation proceeded with a 3 + 3 design from 375 mg to 625 mg of ONC201. One cycle, also the dose-limiting toxicity (DLT) window, was 21 days. The primary endpoint was to determine the RP2D of weekly ONC201, which was confirmed in an 11-patient dose expansion cohort.

**Results:**

Twenty patients were enrolled: three at 375 mg and 17 at 625 mg of ONC201. The RP2D was defined as 625 mg with no DLT, treatment discontinuation, or dose modifications due to drug-related toxicity. PK profiles were consistent with every-three-week dosing and similar between the first and fourth dose. Serum prolactin and caspase-cleaved cytokeratin-18 induction were detected, along with intratumoral integrated stress response activation and infiltration of granzyme B+ Natural Killer cells. Induction of immune cytokines and effectors was higher in patients who received ONC201 once weekly versus once every three weeks. Stable disease of > 6 months was observed in several prostate and endometrial cancer patients.

**Conclusions:**

Weekly, oral ONC201 is well-tolerated and results in enhanced immunostimulatory activity that warrants further investigation.

**Trial registration:**

NCT02250781 (Oral ONC201 in Treating Patients With Advanced Solid Tumors), NCT02324621 (Continuation of Oral ONC201 in Treating Patients With Advanced Solid Tumors).

**Electronic supplementary material:**

The online version of this article (10.1186/s40425-019-0599-8) contains supplementary material, which is available to authorized users.

## Background

Dysregulation of the dopamine pathway has been reported in a range of malignancies that frequently upregulate expression of DRD2, one of the Gi-coupled D2-like dopamine receptors that control ERK signaling and cAMP production [1–5]. ONC201 is an orally active small molecule found to be a selective DRD2/3 antagonist following its phenotypic discovery as a p53-independent anti-cancer compound that inactivates Akt and ERK to cause downstream induction of the TRAIL gene [6–8]. The compound has exhibited sustained pharmacodynamics and antitumor efficacy following single-dose administration in several preclinical models of solid tumors and hematological malignancies [9–11]. Dose intensification experiments in xenograft models revealed that the compound has saturable efficacy in vitro and in vivo with a wide therapeutic window that led to an initial clinical schedule of once every 3 weeks at a target dose of 625 mg in adults.

Outside of neurology and oncology contexts, DRD2 is also expressed on the surface of a variety of immune cells, and dopamine has been implicated in the regulation of immune cell activity [12]. DRD2 antagonism has been reported to induce immune cell proliferation and activation in preclinical studies [13]. Recent preclinical studies with ONC201 in immunocompetent mouse models have found that ONC201 induces the proliferation of NK cells that appear to be activated based on their expression of effector molecules TRAIL and granzyme B [14]. This observation was apparent systemically amongst peripheral blood mononuclear cells and tumor-infiltrating lymphocytes. Furthermore, depletion of NK cells from these mice has been observed to blunt the antitumor efficacy of ONC201, suggesting that immunostimulatory activity may be a component of its overall antitumor mechanism and that immune modulation should be monitored as a pharmacodynamic readout.

We previously conducted a first-in-human dose escalation trial of ONC201 in adult advanced solid tumor patients [15]. This trial did not identify a maximum-tolerated dose for ONC201 and dose escalation was terminated at a dose of 625 mg once every 3 weeks. This was the protocol pre-specified maximum administered dose (MAD) based on preclinical efficacy, PK, and PD results, which indicated that targeted thresholds were surpassed. Given that the MTD was not reached and that the PK profile indicated systemic clearance by 7 days, we sought to evaluate the clinical feasibility of weekly administration of ONC201.

## Methods

### Patients

Eligible patients had an advanced solid tumor that is refractory to standard treatment, or for which no standard therapy is available, or the subject refused standard therapy. Other inclusion criteria included: ≥18 years of age, Eastern Cooperative Oncology Group performance status of 0 or 1, adequate organ and marrow function, and measurable disease by Response Evaluation Criteria in Solid Tumors version 1.1 (RECIST 1.1). The first patient enrolled on the study received the first dose of ONC201 on May 2, 2016.

### Study design and objectives

This was a single-arm, single-agent, single-center, Phase I dose escalation and expansion study that evaluated the safety and tolerability of ONC201 in patients with advanced solid tumors. The primary objective was to determine the RP2D, defined as the MTD or the protocol pre-specified MAD of 625 mg, whichever was achieved. Antitumor activity was assessed using RECIST 1.1. Patients with prostate cancer were assessed according to Prostate Cancer Working Group 2 criteria [16]. Secondary objectives included PK, PD, and antitumor activity by RECIST 1.1.

### Study drug administration

Patients received ONC201 at 375 mg or 625 mg orally once per week, until RECIST 1.1–defined progression occurred. One treatment cycle was defined as 21 days.

### Study assessments

Screening and baseline assessments were obtained within 28 days prior to the first dose of ONC201. After baseline evaluation, objective tumor assessments were performed every two cycles (6 weeks ±7 days) until disease progression. Response to treatment was assessed by RECIST 1.1. Blood samples for PK and PD analyses were collected. The safety and tolerability of ONC201 were assessed according to Common Terminology Criteria for Adverse Events (version 4.03). On-treatment biopsies were obtained from two patients: one with prostate cancer and another with endometrioid cancer.

### Pharmacodynamic (PK) analyses

Plasma samples were prepared from blood collected in K_2_EDTA tubes at baseline, 30 min, 2, 4, 6, 24, 48, and 168 h following the first two cycles of ONC201 and pre-dose on day 1 of subsequent cycles. PK was analyzed, as previously described [15].

### Pharmacodynamic (PD) analyses

Blood samples for PD analyses were collected at pre-dose, 6 h, 2, 3, 8, and 15 days after ONC201 treatment for cycles 1 and 2, and at pre-dose on day 1 for cycles 3 and beyond. Caspase-cleaved cytokeratin 18 (cCK18) was measured as a biomarker of epithelial cell apoptosis and prolactin was measured as a surrogate biomarker of DRD2 antagonism, as previously described (Diapharma; #P10011) [15]. Immunohistochemistry for CHOP (Proteintech; # 5204–1-AP), DR5 (Novus; NB100–56618), CD56 (BioLegend, Catalog X), Granzyme B (Abcam; Catalog X) and TUNEL (Novus; #NBP2–31164) was carried out per manufacturer’s instructions on slides prepared from formalin-fixed paraffin-embedded tissue. Serum was analyzed for expression of cytokines and effector molecules using LegendPlex assay per manufacturer’s instructions (BioLegend; #746267).

### Statistical considerations

As a Phase I study, results were reported using descriptive statistics. The trial size was driven by the standard 3 + 3 design that has a total enrollment that is dependent on the safety experience. An 11-patient expansion cohort was enrolled to provide more precision around the degree of toxicity and PK profile associated with the RP2D. For assessment of the significance of progression-free survival, a log rank hazard ratio calculation was performed for induction of perforin (GraphPad Prism). For pairwise comparisons, a student’s two-tailed t test was performed (Microsoft Excel).

## Results

### Patients and treatment

In total, 20 evaluable patients were enrolled to this trial. During dose escalation, three patients were enrolled in the 375 mg cohort, and six patients were enrolled in the 625 mg cohort. An additional 11 patients were enrolled in the dose expansion cohort who also received 625 mg of ONC201. All patients received at least three doses of ONC201 and thus completed the DLT window. Baseline demographic and clinical characteristics were typical of an adult unselected advanced solid tumor population (Table 1). All patients had prior systemic therapy (predominantly chemotherapy), radiotherapy, and surgery. Tumor types included eight (40%) prostate cancer patients, five (25%) colon cancer patients, four (20%) endometrial cancer patients and three (15%) glioblastoma patients.

**Table 1:** Demographics and clinical characteristics of patients treated with ONC201 who were evaluable for safety and efficacy

	Patients (*N* = 20)	
ONC201 (mg)	375	625
No. of Patients (%)	3 (15%)	17 (85%)
Male	2	8
Female	1	9
ECOG		
ECOG = 0	2 (10%)	6 (30%)
ECOG = 1	1 (5%)	11 (55%)
Age (years)^a^	80 (42–92)	64 (35–79)
Weight (kg)^a^	51.3 (50.4–55.0)	82.7 (56.4–109.1)
Doses^a^	21 (18–30)	19.6 (4–70)
Prior Therapies		
Prior hormonal therapies^a^	0	1.2 (1–6)
Prior chemotherapies^a^	2.3 (0–4)	2.2 (0–7)
Prior immunotherapies^a^	1 (0–2)	0.2 (0–1)
Prior targeted therapies^a^	0.7 (0–2)	0.8 (0–4)
Prior Radiation^a^	1.5 (1–2)	1 (1–3)
Prior Surgery^a^	2 (2–3)	3 (1–7)

^a^ Indicates that the datum is reported as the mean with the range in parentheses

### Safety

No DLT, treatment discontinuation, or dose modifications due to drug-related toxicity occurred in any cohort that was attributed to the study drug (Additional file 1: Table S1). Eight Grade 1 adverse events (AEs) were attributed as possibly related to the study drug. No drug-related Grade > 1 AEs were reported. This included the safety experience during the DLT window and beyond that continued for > 6 months in five patients and one patient who continued therapy for > 50 weeks.

### Pharmacokinetics

**Fig. 1:**
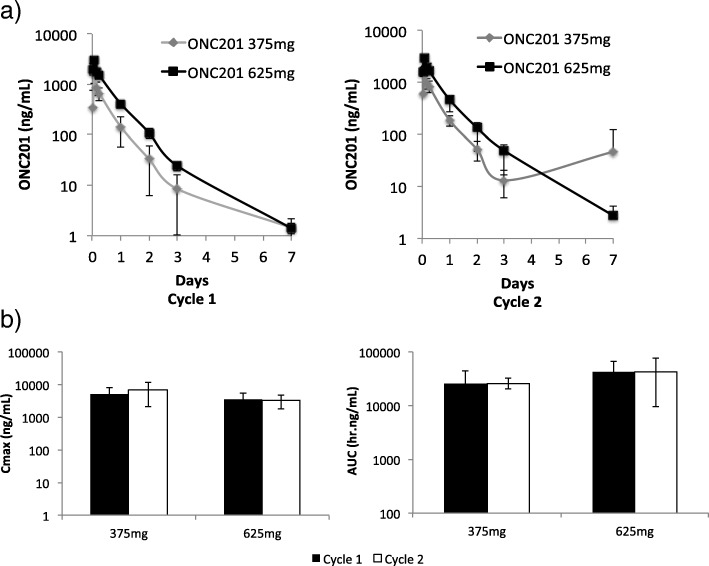
Pharmacokinetic analysis of ONC201. **a** ONC201 plasma concentrations following the first dose of cycle 1 and cycle 2. Concentrations are shown as the mean for each dose cohort in dose escalation (375 mg and 625 mg). **b** Cmax and AUC for 375 mg (*n* = 3) and 625 mg (*n* = 17) dose cohorts for cycle 1 and cycle 2. Each error bar indicates SEM

The PK profile of ONC201 during cycle 1 was consistent with previous results from the once every-three-week schedule (Fig. 1) [15]. Expected PK parameters were achieved and include a 4.3 μg/mL Cmax, 34.3 h*μg/mL AUC, and 9.4 h half-life. Comparisons of the PK profile from the first day of cycle 1 (after the first dose of ONC201) and cycle 2 (after the fourth dose of ONC201) revealed similar profiles with no significant differences in PK parameters (Fig. 1b and Additional file 1: Table S2). Thus, weekly dosing of ONC201 did not appear to result in systemic accumulation or altered metabolism that altered its previously reported PK profile.

### Pharmacodynamics

**Fig. 2:**
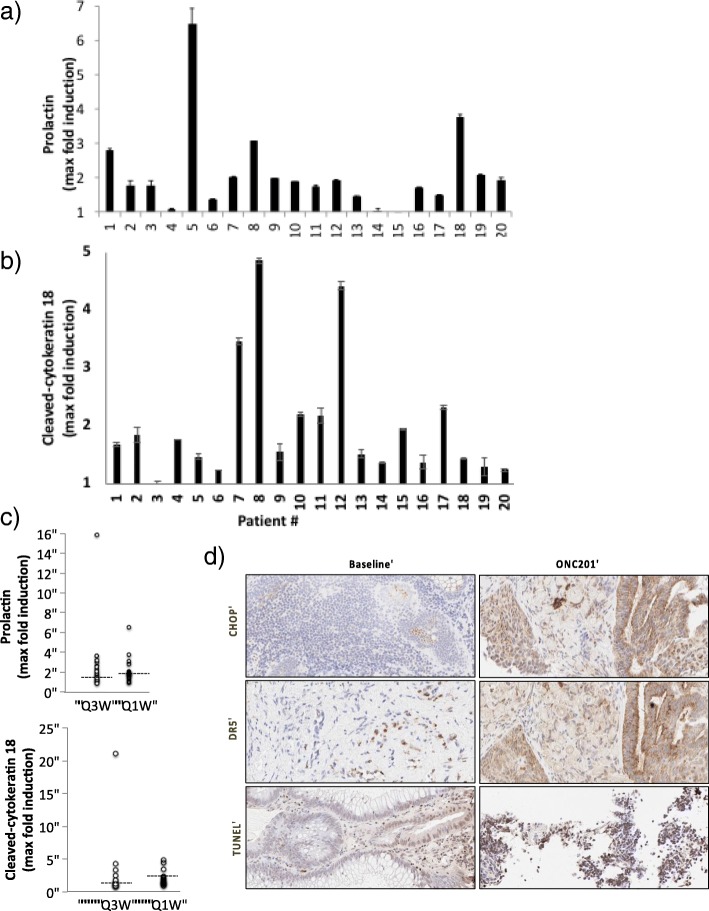
Pharmacodynamic assays for ONC201. Maximum fold induction of serum prolactin levels (**a**) and cleaved cytokeratin 18 (**b**) in patients relative to baseline levels. **c** Max fold induction of prolactin and cleaved cytokeratin 18 in patients treated at once every three weeks (Q3W) and on the weekly (Q1W) schedule relative to baseline levels. Median prolactin levels: 1.79 and 1.84 for Q3W and Q1W cohorts, respectively. Median cleaved cytokeratin levels: 1.25 and 1.61 for Q3W and Q1W cohorts, respectively. **d** IHC analyses of CHOP, DR5, and double-stranded DNA breaks (TUNEL) in baseline and ONC201-treated biopsies for an endometrial cancer patient. The biopsy was done 9.8 months after starting ONC201 treatment (7 days after the most recent ONC201 dose). Each error bar indicates SEM

Serum prolactin was evaluated as a surrogate biomarker of DRD2 antagonism [17]. Consistent with the prior Phase I experience, the majority of patients exhibited a > 2-fold induction of prolactin (Fig. 2a). More than 75% of patients had at least a 50% induction. Induction of serum caspase-cleaved cytokeratin 18 as a biomarker of epithelial cell apoptosis was also observed in 65% of the evaluable patients (Fig. 2b and Additional file 1: Figure S1). The magnitude of induction of these pharmacodynamic biomarkers was similar between the two dose schedules (Fig. 2c). There was no obvious association of either of these serum pharmacodynamic biomarkers with systemic exposure to ONC201 (Additional file 1: Figures S3 and S4) or clinical outcomes. Nevertheless, these PD results supported the previous assertion that 625 mg is a biologically active dose of ONC201.

ONC201 is known to activate the integrated stress response pathway that is associated with induction of CHOP and DR5, leading to apoptosis, and has been shown to mediate the activity of ONC201 in preclinical models [18]. One paired biopsy was obtained on this study from a metastatic lymph node of a patient with metastatic endometrial cancer one week prior to initiating ONC201 (baseline biopsy) and 9.8 months after beginning ONC201 (immediately before the 43rd dose). Comparing the baseline to on-treatment biopsy, ONC201 treatment increased the expression of CHOP and DR5 (Fig. 2d). Increased tumor cell apoptosis was also apparent in the biopsy, as evidenced by TUNEL staining (Fig. 2d). The patient continues on ONC201 for > 50 weeks with stable disease by RECIST and continued on therapy without drug-related adverse events.

### Immune modulation

**Fig. 3:**
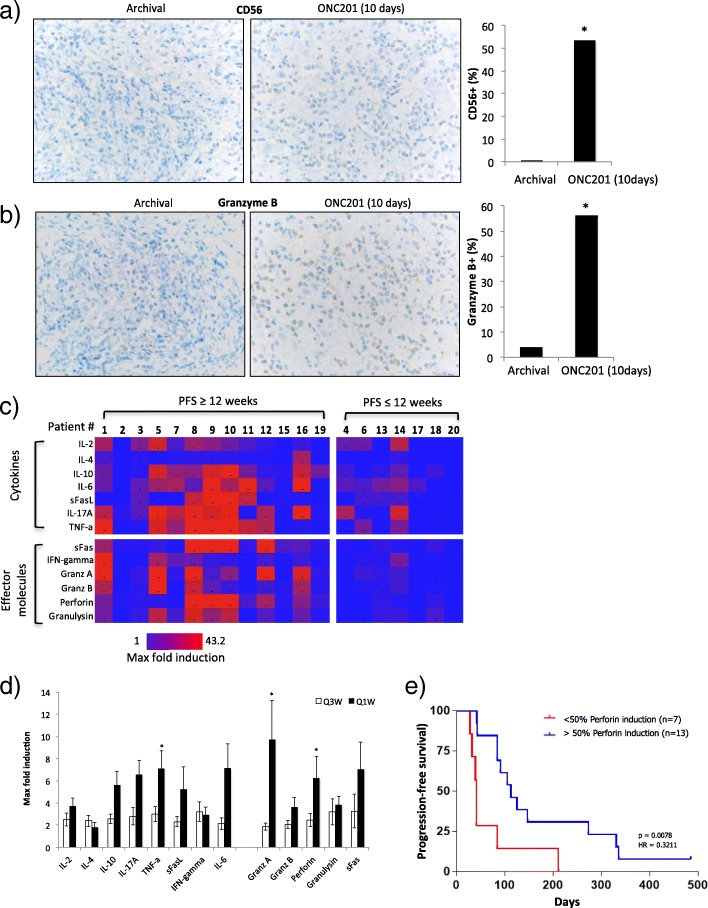
Immunostimulatory activity of ONC201. Immunohistochemical analysis of CD56+ (**a**) and granzyme B+ (**b**) cells in archival and post-ONC201 biopsy tumor tissue of an enzalutamide-refractory prostate cancer patient. Positive staining is depicted in gray color (*P* < 0.05). * denotes *P* < 0.05 by student’s two-tailed t test comparing the on-treatment tissue staining to archival tissue staining. **c** Heat map depicting maximum fold induction relative to baseline of immune cytokines and effector molecules in patients with PFS ≥ 12 weeks versus PFS < 12 weeks. * denotes *P* < 0.05 by student’s two-tailed t test comparing the on-treatment tissue staining to archival tissue staining. **d** Maximum fold change over baseline of immune cytokines and effector molecules in patients with PFS > =12 weeks by RECIST who received ONC201 at various dose levels once every three weeks or once weekly. Each error bar indicates SEM. * denotes *P* < 0.05 by student’s two-tailed t test comparing the maximum values to the baseline value. **e** PFS in patients who had at least a 50% induction in perforin following ONC201 once every three weeks or once weekly (*P* = 0.0078; HR 0.3211)

One additional patient, who had metastatic prostate cancer, underwent an on-treatment lymph node biopsy that was compared to archival tumor tissue from the same lymph node. Comparisons revealed increased infiltration of granzyme B+ and CD56+ cells, corroborating preclinical reports of NK cell intratumoral infiltration in response to ONC201 (Fig. 3a-b). Limited tissue availability precluded interrogation of this patient’s on-treatment biopsy for additional intratumoral PD, such as integrated response activation.

Since tumor biopsies were not available for most patients, immune cytokine and effector profiling was conducted from serum samples using a multiplex cytokine assay (Fig. 3c). A broad induction of immune cytokines and effector molecules was observed amongst patients treated with ONC201, in particular among patients who experienced at least stable disease by RECIST for 12 or more weeks. Examining the kinetics of this response revealed that maximum immune cytokine induction tended to occur within the first two cycles, while maximum effector induction tended to occur beyond cycle 2 (Additional file 1: Figure S5). Examination of the serum samples from previously reported patients treated on the once every-three-weeks schedule revealed a similarly broad immune cytokine and effector molecule induction (Fig. 3d). The magnitude of induction was generally lower than that of patients who received ONC201 once weekly, though this did not reach statistical significance (Additional file 1: Figure S2). Patients who had a > 50% induction in serum perforin, a cytolytic protein found in granules of cytotoxic T lymphocytes (CTLs) and NK cells [19], upon ONC201 administration had a significantly longer progression-free survival (*P* = 0.078; HR = 0.3211) (Fig. 3e).

### Radiographic tumor evaluation

**Fig. 4:**
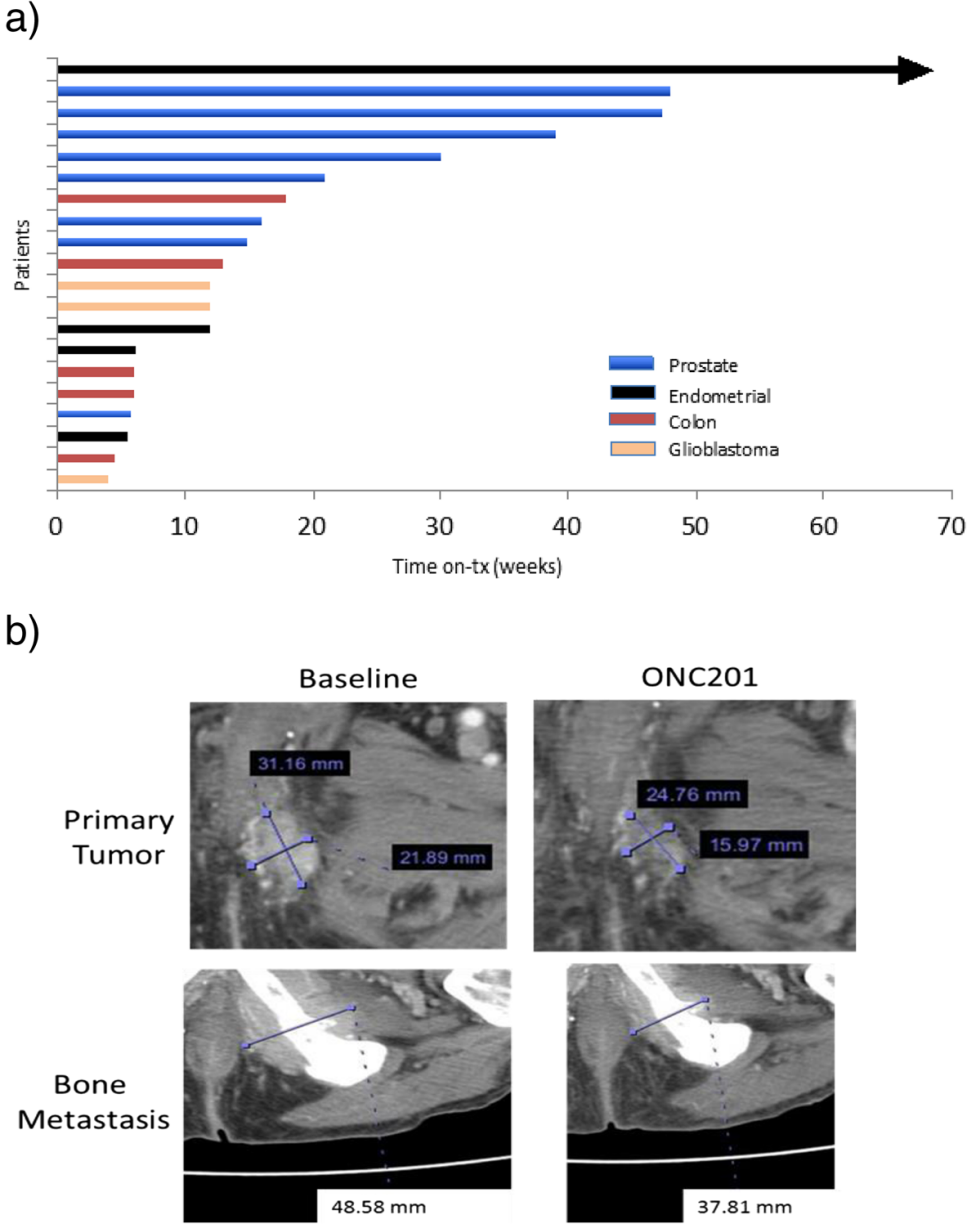
Clinical outcome of ONC201-treated patients. **a** Swimmer plot of ONC201-treated patients showing progression-free survival, as defined by RECIST version 1.1. Each bar represents one patient on the study who has been treated on a weekly schedule with ONC201. Arrowhead indicates that the patient was still on treatment at the time of writing this manuscript. **b** Primary tumor and bone metastasis measurements from a patient with prostate cancer at baseline and after two doses of weekly 625 mg ONC201

A disease-control rate of 42.9% was achieved amongst all patients. Prolonged stable disease for > 6 months by RECIST criteria was observed in five (23.8%) patients (Fig. 4a). Similar to the prior Phase I experience, regressions in individual metastatic lesions were noted in prostate and endometrial cancer patients. One heavily pre-treated endometrioid cancer patient remained progression-free on treatment for > 70 weeks. Four enzalutamide-resistant prostate cancer patients had PFS > 30 weeks. One prostate cancer patient experienced significant shrinkage (> 20%) by RECIST criteria in primary tumor and bone metastasis after two doses (625 mg) of ONC201 (Fig. 4b). Prostate cancer patients who exhibited prolonged stable disease had stabilized or modest increases in PSA over time, unlike patients who progressed rapidly (Additional file 1: Figure S6).

An enzalutamide-refractory metastatic prostate cancer patient with lymph node involvement exhibited intratumoral infiltration of activated NK cells and had a 70% induction of serum caspase-cleaved cytokeratin-18 72 h after the first dose of 625 mg ONC201. This systemic and intratumoral PD activity was accompanied by prolonged stable disease for 48 weeks.

Consistent with the observation that immune effector induction is associated with patients who experienced prolonged stable disease, progression-free survival was superior in patients who experienced immune effector induction after beginning ONC201. The three patients with the longest PFS also had > 1.8-fold induction of cleaved-cytokeratin 18 (Fig. 2b) and induction of immune cytokines and effectors (Fig. 3c).

## Discussion and conclusions

This is the first report of ONC201 administered on a weekly dosing schedule to humans. The safety and tolerability of ONC201 has continued to prove exceptional at doses that exceed the targeted PK and PD profiles. Importantly, the PK parameters such as Cmax in the top dose cohort (625 mg) were not significantly different from 375 mg cohort of patients and exceeded the concentration associated with the antitumor efficacy in mouse models and the NOAEL in toxicology studies. Serum prolactin levels in the patients indicated that ONC201 engaged its target in advanced cancer patients at, and below, its RP2D dose of 625 mg that is 5-fold above the reported efficacious dose of 25 mg/kg in murine models [15]. These results indicate that ONC201 is equally well tolerated in adult advanced cancer patients on a once-weekly versus every-three-weeks schedule at a dose of 625 mg. In addition to clinical feasibility, the finding that weekly ONC201 produced a stronger systemic immune response adds to the rationale for this administration schedule. While weekly dosing of ONC201 appeared to induce the desired PD, more frequent dose schedules are being evaluated in acute leukemias (NCT02392572).

The clinical validation of the immunostimulatory activity of ONC201 adds another dimension of activity to be considered for this investigational agent. The late immune effector induction that was associated with patients who experienced prolonged stable disease should be further investigated, as patients with progressive disease did not have additional blood sampling after coming off protocol. Therefore, it is not known if patients with progressive disease would have also experienced immune effector induction at later time points and whether or not that would influence outcome as a late response. The benign safety profile of ONC201 could enable future clinical trials to treat beyond radiographic progression, which is increasingly implemented for immunotherapies due to delayed treatment benefit and radiographic pseudo-progression resulting from intratumoral infiltration by immune cells. This has been further supported for ONC201 by the intratumoral NK cell infiltration observed in the patient biopsied on this trial and in recently reported preclinical studies [20].

Despite the enrollment of an advanced cancer population that was heavily pretreated and unselected, the disease control rate and prolonged stable disease was encouraging. Given that these observations occurred in prostate and endometrial cancer patients in both the every-three-week and every-one-week Phase I experience [15], further clinical investigation in these tumor types is warranted. Phase II trials to evaluate the efficacy of ONC201 in recurrent/refractory metastatic endometrial cancer are ongoing (NCT03099499, NCT03485729).

Tumor cells utilize a range of mechanisms during oncogenesis that allow them to activate pro-survival signaling pathways. Genetic alterations such as point mutations and gene fusions that result in activation of oncogenes and inactivation of tumor suppressors have represented a category of well-established therapeutic targets. However, observations that tumors coopt their microenvironment to accomplish similar phenotypes have not been as widely translated into therapeutic approaches that capitalize on the dependency of tumor cells on these factors. Within this area, the ability of tumor cells to dysregulate neurotransmission pathways such as dopamine receptor signaling to control pro-survival and stress signaling pathways has emerged in gliomas and other malignancies [21]. Dopamine receptors such as DRD2 are often overexpressed by tumor cells and can be activated by dopamine produced in its microenvironment or by the tumor cells themselves. Preclinical studies have shown that antagonism of DRD2 to shut off this paracrine and/or autocrine pro-survival signaling pathway induces tumor cell apoptosis via inactivation of Akt and ERK, among other anti-cancer signaling consequences [1, 4, 5].

DRD2 antagonism using genetic or pharmacological methods has been shown to produce antitumor effects in several preclinical cancer models; however, antipsychotic DRD2 competitive antagonists have shown an inferior tolerability and anti-cancer efficacy that is likely due to their pleiotropy amongst dopamine receptors and other GPCRs under physiological conditions [1, 4]. In contrast, ONC201 is both a competitive and non-competitive antagonist that only engages DRD2 and its closely related family member DRD3 at physiological concentrations. These unique features likely underpin the exceptional safety profile of ONC201 that continues to be observed in clinical trials.

It is important to consider our findings reported here in the context of the limitations of our study, including the limited number of patients enrolled and specimens available for analysis and the heterogeneity of the enrolled patients, which preclude us from making global conclusions based on anecdotal data. However, we expect to verify our findings from further clinical investigation of ONC201 using the weekly regimen established in this trial, and important observations are expected to be made in ongoing clinical trials focusing on tumor types that are dependent on dopamine receptor dysregulation, such as high-grade gliomas, where ONC201 has shown compelling preclinical efficacy and encouraging early evidence of antitumor activity.

## Additional file

Additional file 1: **Figure S1.** Ratio of cleaved:total cytokeratin 18 (M30/M65 ELISA assay) in patients treated with weekly ONC201. **Figure S2.** Maximum fold change over baseline of immune cytokines and effector molecules in all ONC201-treated patients in the two dosing cohorts (every three weeks and weekly dosing schedules). **Figure S3.** (A) Maximum fold change of serum prolactin levels in the serum relative to baseline when compared to maximum concentration of ONC201 in the serum of the patients treated on a weekly schedule. **Figure S4.** Maximum fold induction of caspase-cleaved cytokeratin 18 levels in the serum relative to baseline when compared to maximum concentration of ONC201 in the serum of the patients treated on a weekly schedule. **Figure S5.** Timing of maximum fold-induction of immune cytokines and effects. **Figure S6.** Serum PSA (ng/mL) of ONC201-treated prostate cancer patients. **Table S1.** Treatment-related adverse events (AEs) in patients treated with ONC201 on a weekly schedule. **Table S2.** Pharmacokinetic parameters for 625 mg of ONC201 after the first dose of cycle 1 and after the first dose of cycle 2 (*n* = 17). (DOCX 324 kb)
