# Supplementary material for: Safety and enhanced immunostimulatory activity of the DRD2 antagonist ONC201 in advanced solid tumor patients with weekly oral administration
Source: J Immunother Cancer. 2019 May 22;7:136. doi: 10.1186/s40425-019-0599-8 (PMC6532211; doi:10.1186/s40425-019-0599-8)
Supplement: Supplementary file 1 — Figure S1. Ratio of cleaved:total cytokeratin 18 (M30/M65 ELISA assay) in patients treated with weekly ONC201. Figure S2. Maximum fold change over baseline of immune cytokines and effector molecules in all ONC201-treated patients in the two dosing cohorts (every three weeks and weekly dosing schedules). Figure S3. (A) Maximum fold change of serum prolactin levels in the serum relative to baseline when compared to maximum concentration of ONC201 in the serum of the patients treated on a weekly schedule. Figure S4. Maximum fold induction of caspase-cleaved cytokeratin 18 levels in the serum relative to baseline when compared to maximum concentration of ONC201 in the serum of the patients treated on a weekly schedule. Figure S5. Timing of maximum fold-induction of immune cytokines and effects. Figure S6. Serum PSA (ng/mL) of ONC201-treated prostate cancer patients. Table S1. Treatment-related adverse events (AEs) in patients treated with ONC201 on a weekly schedule. Table S2. Pharmacokinetic parameters for 625 mg of ONC201 after the first dose of cycle 1 and after the first dose of cycle 2 (n = 17). (DOCX 324 kb) [file 40425_2019_599_MOESM1_ESM.docx]

**Safety and Enhanced Immunostimulatory Activity of the DRD2 Antagonist ONC201 in Advanced Solid Tumor Patients with Weekly Oral Administration**

Mark N. Stein, MD^1^, Jyoti Malhotra,MD^1^, Rohinton S. Tarapore, PhD^2^, Usha Malhotra, MD^1^, Ann W. Silk, MD^1^, Nancy Chan, MD^1^, Lorna Rodriguez, MD, PhD^1^, Joseph Aisner, MD^1^, Robert D. Aiken, MD^1^, Tina Mayer, MD^1^, Bruce G. Haffty, MD^1^, Jenna H. Newman, AB^1^, Salvatore M. Aspromonte, BS^1^, Praveen K. Bommareddy, MS^1^, Ricardo Estupinian, MS^1^, Charles B. Chesson, PhD^1^, Evita T. Sadimin, MD^1^, Shengguo Li, PhD^1^, Daniel J. Medina, PhD^1^, Tracie Saunders, RN, MS^1^, Melissa Frankel^1^, Aparna Kareddula^1^, Sherrie Damare^1^, Elayne Wesolowsky^1^, Christian Gabel^1^, Wafik S. El-Deiry, MD, PhD^3^, Varun V. Prabhu, PhD^2^, Joshua E. Allen, PhD^2^, Martin Stogniew, PhD^2^, Wolfgang Oster, MD, PhD^2^, Joseph R. Bertino, MD^1^, Steven K. Libutti, MD^1^, Janice M. Mehnert, MD^1^, and Andrew Zloza, MD, PhD^1,4^

**Supplemental Materials**

**Supplemental Figure Legends**

**Figure S1:** Ratio of cleaved:total cytokeratin 18 (M30/M65 ELISA assay) in patients treated with weekly ONC201. Patients 1-3 received 375 mg of ONC201 while the remaining patients received 625 mg of ONC201. Each error bar indicates SEM.

**Figure S2:** Maximum fold change over baseline of immune cytokines and effector molecules in all ONC201-treated patients in the two dosing cohorts (once every three weeks and weekly dosing schedules). Each error bar indicates SEM.

**Figure S3:** (A) Maximum fold change of serum prolactin levels in the serum relative to baseline when compared to maximum concentration of ONC201 in the serum of the patients treated on a weekly schedule.

**Figure S4:** Maximum fold induction of caspase-cleaved cytokeratin 18 levels in the serum relative to baseline when compared to maximum concentration of ONC201 in the serum of the patients treated on a weekly schedule.

**Figure S5:** Timing of maximum fold-induction of immune cytokines and effects. Each dot represents the time when maximum induction was observed for the top five patients who had the highest immune induction.

**Figure S6:** Serum PSA (ng/mL) of ONC201-treated prostate cancer patients.

**Supplemental Figures**

**Table S1:** Treatment-related adverse events (AEs) in patients treated with ONC201 on a weekly schedule. Those AEs attributed that are at least possibly related to the study drug are listed and all are Grade 1.

| **No. of Patients** | 20 |
| --- | --- |
| Nausea | 1 (5%) |
| Vomiting | 1 (5%) |
| Fatigue | 2 (10%) |
| Stomach Pain | 1 (5%) |
| Cognitive Disturbance | 1 (5%) |
| Tinnitus | 1 (5%) |
| Dysgeusia | 1 (5%) |

**Table S2:** Pharmacokinetic parameters for 625 mg of ONC201 after the first dose of cycle 1 and after the first dose of cycle 2 (n = 17).
